# Tetracycline Removal by Hercynite-Biochar from the Co-Pyrolysis of Red Mud-Steel Slag-Sludge

**DOI:** 10.3390/nano12152595

**Published:** 2022-07-28

**Authors:** Xian Zhou, Xia Chen, Wei Han, Yi Han, Mengxin Guo, Ziling Peng, Zeyu Fan, Yan Shi, Sha Wan

**Affiliations:** 1 Changjiang River Scientific Research Institute, Research Center of Water Engineering Safety and Disaster Prevention of Ministry of Water Resources, Wuhan 430010, China; zhouxian@mail.crsri.cn (X.Z.); chenxia@mail.crsri.cn (X.C.); hanwei@mail.crsri.cn (W.H.); pengziling0304@163.com (Z.P.); fanzeyu717728@163.com (Z.F.); 18163559730@163.com (Y.S.); 18607217399@163.com (S.W.); 2 College of Resources and Environment, Anqing Normal University, Anqing 246011, China; gmx7317653@163.com

**Keywords:** FeAl_2_O_4_, biochar, dewaterability, tetracycline, adsorption

## Abstract

The sludge-derived biochar is considered an effective emerging contaminants adsorbent for wastewater treatment. In this paper, red mud and steel slag (RMSS) was used for improving sludge dewaterability and enhancing the sludge-derived biochar adsorption capacity. X-ray diffraction (XRD), Fourier transform infrared spectroscopy (FTIR), and a scanning electron microscope (SEM) were employed to comprehensively characterize the mineral composition, functional group, and morphology of the adsorbent. RMSS was able to improve the sludge dewatering performance by providing a skeleton structure to promote drainage and Fe(III) to decrease the Zeta potential. The dosage of 20 mg/g RMSS was able to reduce the specific resistance to filtration (SRF) and the Zeta potential of sludge from 1.57 × 10^13^ m/kg and −19.56 mV to 0.79 × 10^13^ m/kg and −9.10 mV, respectively. The co-pyrolysis of RMSS and sludge (2:8) induced the formation of biochar containing FeAl_2_O_4_ (PS80). The PS80 exhibited a large surface area (46.40 m^2^/g) and high tetracycline (TC) removal capacity (98.87 mg/g) when combined with H_2_O_2_ (PS80-H_2_O_2_). The adsorption process of TC onto PS80 and PS80-H_2_O_2_ was well described by the pseudo-first-order and pseudo-second-order kinetic model, indicating physisorption and chemisorption behavior. The results indicated that co-pyrolysis of RMSS sludge PS80-H_2_O_2_ could enhance the biochar adsorption capacity of TC, attributable to the degradation by ·OH generated by the heterogeneous Fenton reaction of FeAl_2_O_4_ and H_2_O_2_, the release of adsorbed sites, and the improvement of the biochar pore structure. This study proposed a novel method for the use of RMSS to dewater sludge as well as to induce the formation of FeAl_2_O_4_ in biochar with effective TC removal by providing a Fe and Al source, achieving a waste-to-resource strategy for the integrated management of industrial solid waste and sewage sludge.

## 1. Introduction

As the end-of-life product of the domestic sewage treatment process, sewage sludge has had an increase in production year by year and contains a variety of pollution components, which have attracted much attention [1]. At present, the disposal methods of sludge are mainly landfilling, incineration, and pyrolysis [2]. Among them, landfilling and incineration have a high risk of producing secondary pollution and cannot acquire resource utilization [3]. Sludge pyrolysis can eliminate pollution components such as pathogens and obtain biochar materials at the same time, so it is considered to have good application prospects [4,5]. Sludge dewatering, as a treatment method before sludge is transported out of the treatment plant, determines the water content of the sludge cake. The water content of sludge cake treated by traditional dewatering methods is as high as approximately 80% [6], which is not conducive to subsequent pyrolysis disposal. In recent years, scholars have studied various means, such as the use of microwave, hydrothermal, and chemical conditioning, to achieve the deep dewatering of sludge [7,8,9]. Previous studies have confirmed that steel slag and red mud can help improve sludge dewatering performance [10,11,12], but there are few studies on the pyrolysis of sludge cake containing large amounts of iron–aluminum oxides.

The conversion of biomass to carbonaceous materials is being considered as an alternative to solid waste management [13]. Pyrolysis sludge biochar is often used to adsorb heavy metals or organic pollutants, and it has also been acknowledged that iron-rich biochar can catalyze H_2_O_2_, which triggers the Fenton or Fenton-like catalytic oxidative degradation of various organic pollutants, especially antibiotics [14,15,16]. The iron-containing phase of sludge-derived biochar is complicated. Apart from the generation of iron oxides with different valence states, iron forms ferrites with other elements during the thermal treatment process [17,18]. Therefore, scholars have proposed that the catalytic effect of Fe-rich biochar is mainly realized by heterogeneous Fenton induced by iron-bonded and homogeneous Fenton-induced leached Fe^2+^ from Fe-rich biochar [19,20]. The phase transformations of Fe and Al compounds in sludge-derived biochar during sludge pyrolysis after adding both Fe_2_O_3_ and Al_2_O_3_ were studied by Tao et al. [21], confirming that the FeAl_2_O_4_ phase was formed in sludge biochar with a good level of catalytic performance. Heterogeneous catalytic activities were also demonstrated in phenol degradation [22]. Red mud and steel slag (RMSS) is rich in iron and aluminum and has the potential for being used in the preparation of biochar containing FeAl_2_O_4_ phase sludge, but there is little research on this. Therefore, the objective of this experimental study was to characterize the effect of the blend ratio of the RMSS and sewage sludge on the co-pyrolysis products and the adsorption performance and mechanism of the hercynite-biochar.

In this study, steel slag modified by salicylic acid and red mud modified by citric acid were used as conditioners to obtain sludge cake rich in iron–aluminum oxides after dewatering. The influence of the proportion of steel slag and red mud in sludge cake on the formation of the FeAl_2_O_4_ phase was studied, and the formation mechanism was revealed by combining the XRD quantitative analysis. In addition, the degradation of tetracycline by H_2_O_2_ catalyzed by sludge biochar was studied, and the catalytic performance of FeAl_2_O_4_ was confirmed. It is hoped that this study can provide guidance for the resource utilization of sludge cake conditioned by steel slag and red mud.

## 2. Materials and Methods

### 2.1. Materials

The raw sludge (RS) was collected from the secondary sedimentation tank of the municipal wastewater treatment plant in Anqing, China. The RS was subsequently stored in a refrigerator at 4 °C before testing, and all the dewatering tests were completed within one week. The following were the main characteristics of the sludge: moisture content (Mc), 96.33%; total solid (TS), 35,366.7 ± 89.7 mg/L; volatile solid (VS), 11,575.0 ± 45.1 mg/L; pH value, 7.03 ± 0.09; SRF, 1.57 ± 0.0 9 × 10^13^ m/kg; Wc of dewatered sludge cake, 81.64 ± 0.71%. The steel slag (SS) was obtained from the Wuhan Iron and Steel Corporation in China. The red mud (RM) originated from the Shan Dong branch of the Aluminum Corporation of China and was a residue of bauxite treated using the Bayer process. The chemical and mineral compositions of SS, RS, and RM were analyzed by X-ray fluorescence, as shown in Table 1. C_7_H_6_O_3_ (AR, 99.5%), C_6_H_8_O_7_·H_2_O (AR, 99.5%), H_2_O_2_ (AR, 30 wt%), and HCl (AR, 37%) were obtained from the Sinopharm Chemical Reagent Company, China. Tetracycline standard compound (>95% purity) was obtained from the Aladdin Chemical Company (Shanghai, China).

### 2.2. Sludge Conditioning with Red Mud and Steel Slag

After drying and crushing, the red mud and steel slag were pretreated and screened through 200 mesh to obtain the products under the sieve. The modified red mud was obtained by adding 0.5 kg red mud powder into 1.5 L salicylic acid solution (0.034 mol/L) and soaking for 10h; the modified steel slag was obtained by adding 0.8kg steel slag powder into 1.5 L citric acid solution (0.034 mol/L) and soaking for 12 h. The modified red mud and steel slag were freeze-dried in a vacuum and then mixed according to the mass ratio of 1:1 to obtain the conditioner RMSS. The sludge conditioning process refers to our previous study [12], The dosage amounts of RMSS are 10, 20, 30, 40, and 50 mg/g of dry sludge (DS). The specific resistance to filtration (SRF) and the water content of sludge cakes (Wc) were used to evaluate the dewatering performance of the sludge. The Zeta potential and the SEM analysis were used to further explain the conditioning mechanism, in which the zeta potential was tested using a Malvern Zetasizer Nano ZS (Malvern Instruments Ltd., UK) by collecting the supernatant of the sludge after centrifugation at 4500 rpm for 5 min before the supernatant was collected and mixed with the sludge at a ratio of 9:1.

### 2.3. Preparation of Biochar Co-Pyrolysis of Sludge and Solid Wastes

The dewatering sludge cake was dried at 105 °C for 12 h. Then, the cake was ground into a powder using a YXOM-0.4L planetary ball mill and screened through a 0.075 mm screen. Then, RMSS was added to the five powder samples, the proportion of sludge mass (DS) in the mixtures were 100%, 80%, 60%, 33%, and 0, respectively. The mixture was ground again in a planetary ball mill at 400r/min for 1 h before pyrolysis. Pyrolysis was performed in an SGL-1400C vacuum tube furnace; 2.0 g from the samples were placed in a quartz boat with nitrogen as the carrier gas. The temperature was gradually increased to 900 °C at a rate of 10 °C/min and maintained for 2 h. Post cooling, the biochar products (PS100, PS80, PS60, PS33, and PS0) were stored in a desiccator for further experiments.

### 2.4. Removal of Tetracycline

The degradation capacities of PS100, PS80, PS60, PS33, and PS0 were characterized by their removal efficiency for tetracycline. Each experiment was conducted by placing 0.08 g biochar at a mass ratio of 1:1 combined with H_2_O_2_ in a glass bottle containing 200 mL tetracycline solution, with an initial concentration of 20 mg/L at 298 K on a shaker at 200 rpm for 24h. Tetracycline removal was determined using an ultraviolet-visible spectrometer at 356 nm [23]. The removal rate was calculated as follows:

*η* = (*C_0_* − *C_t_*)/*C_0_*, (1)

where *C_t_* and *C*_0_ (mg/L) represent the concentrations for tetracycline at time *t* and initially, respectively.

### 2.5. Analysis Method

#### 2.5.1. XRD Rietveld Refinement

The biochar powder extracted in inner fragments and blended with 50% CaF_2_ by mass was characterized by an XRD fine scan (D8 Advance, Karlsruhe, Germany) with a speed of 0.5° per 1 min. The crystal phases in biochar were calculated by MAUD software.

#### 2.5.2. FTIR Analysis

The surface functional groups of biochar before and after the sorption of TC were tested with a Fourier transform infrared (FTIR) spectrometer in the 400–4000 cm^−1^ range (Spectrum 100, PerkinElmer Ltd., Bucks, UK).

#### 2.5.3. Microstructure of Biochar

The microstructure of biochar was observed by scanning electron microscopy (SEM, JSM-5610LV, Electron Optics Laboratory, Tokyo, Japan) after coating with Au for 2 min at 15 mA and 1 mbar. The specific surface areas of the specimens were determined by the Brunauer–Emmett–Teller (BET, BELSORP-mini, Osaka, Japan) method.

## 3. Results and Discussion

### 3.1. Sludge Dewatering Performance

#### 3.1.1. Sludge Dewatering Performance after Conditioning with Solid Wastes

**Figure 1:**
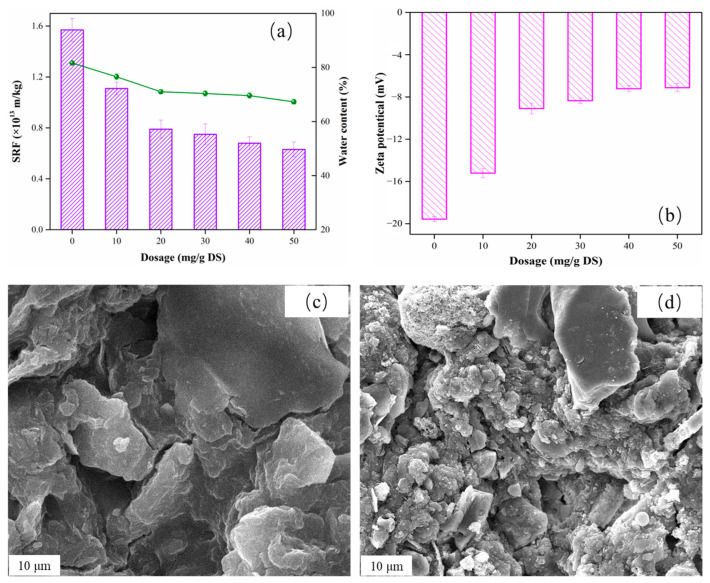
The effects of the RMSS dosage on the conditioning of sludge: (**a**) SRF and Wc; (**b**) zeta potential; SEM images of (**c**) raw sludge and (**d**) the conditioned sludge by RMSS (20% mg/g DS).

The dewatering performance values for the sludge samples conditioned with different RMSS dosages are shown in Figure 1. Compared with RS, the SRF values for the five sludge samples after RMSS conditioning decreased from 1.57 × 10^13^ m/kg to 1.11 × 10^13^ m/kg, 0.79 × 10^13^ m/kg, 0.75 × 10^13^ m/kg, 0.68 × 10^13^ m/kg, and 0.63 × 10^13^ m/kg, respectively. This implies that the dewatering performance of the sludge was effectively improved. When the RMSS dosage increased from 0 mg/g DS to 20 mg/g DS, the sludge dewatering performance decreased significantly. With an increase in the dosage, the decreasing trend becomes insignificant, indicating that excessive RMSS has little effect on the improvement of the sludge dewatering performance. The change in the tendency of the Wc was similar to that for the SRF. The water content values for the five sludge samples conditioned by RMSS were 76.55 wt%, 71.01 wt%, 70.37 wt%, 69.59 wt%, and 67.27 wt%, respectively. When the dosage was more than 20 mg/g DS, the decreasing trend for Wc became slow. When the dosage level for the mud cake reached 50 mg/g DS, the Wc of the sludge cake reached its lowest value, which may be caused by an increase in inorganic content in mud cake. Therefore, in consideration of these findings and of the actual cost, the RMSS dosage of 20 mg/g DS was selected as the best choice.

#### 3.1.2. The Improvement Mechanism for Sludge Dewatering Performance

Extracellular polymer is the most important factor affecting sludge dewatering performance, and zeta potential is significantly related to extracellular polymer [24,25]. Moreover, there is a strong correlation between SRF and zeta potential, which means that zeta potential can better explain the change in sludge dewaterability [26]. The effects of the RMSS dosage on the zeta potential are shown in Figure 1b. The Zeta potential of raw mud is -19.56 mV, which is because there are numerous negatively charged substances in sludge flocs [27]. The existence of a negative charge in the sludge floc was a disadvantage to the flow of water, resulting in poor sludge dewatering performance [28]. After RMSS conditioning with a different dosage, the Zeta potential for each of the five samples decreased to −15.21 mV, −9.10 mV, −8.35 mV, −7.22 mV, and −7.13 mV respectively. The results show that the modified red mud and steel slag was able to effectively reduce the negative charge on the surface of the sludge flocs. This is mainly because there are many iron oxides in red mud and steel slag, which form Fe^3+^ in the conditioning process and play a role in flocculation and charge neutralization. In addition, the modified steel slag can bond with the O-H, C=O, C=C, and N-H in the protein and polyaccharide of the sludge flocs, which reduces the surface charge of the floc particles [29].

The microstructure of the sludge cake before and after conditioning is shown in Figure 1c. The RS had a dense structure and almost no pore distribution on the surface, indicating poor dewaterability. The flocculent structure of the RS was destroyed after being conditioned by RMSS, forming a large granular structure. Next, some irregular massive structure appeared, which acted as a skeleton structure in the extrusion dehydration process, which is conducive to water removal. This is consistent with our previous findings [11].

### 3.2. Effect of Red Mud and Steel Slag Content on the Phase Composition of Sludge Biochar

**Figure 2:**
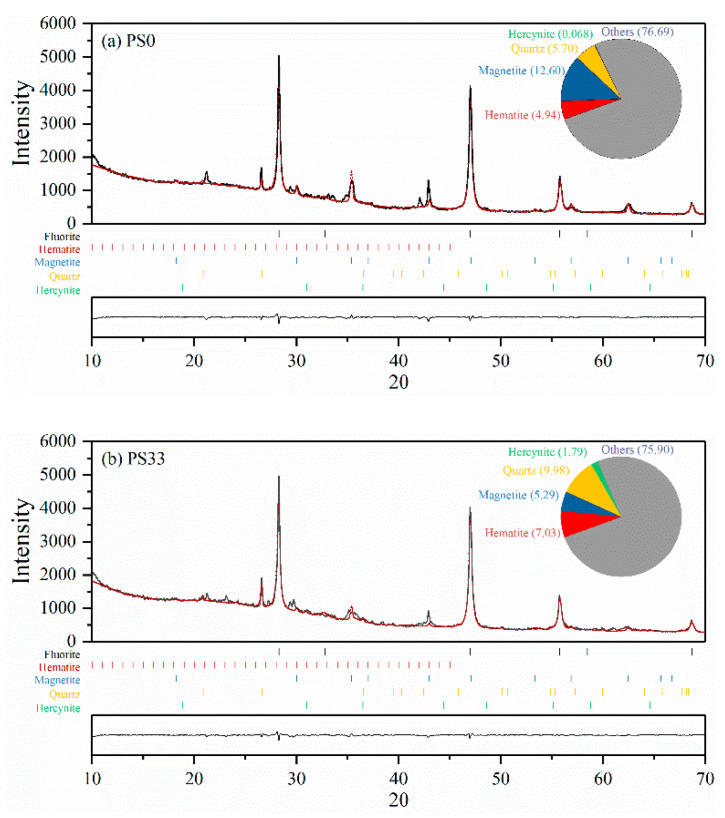

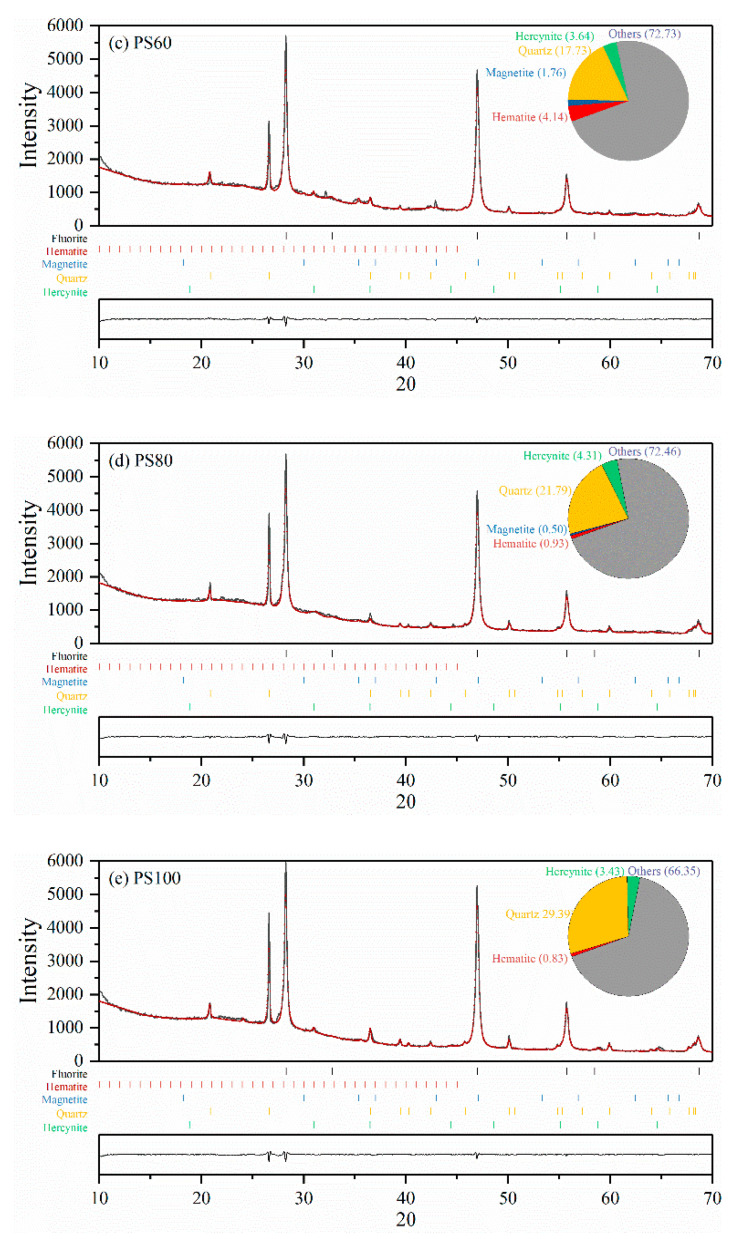
Rietveld refinement of (**a**) PS0, (**b**) PS33, (**c**) PS60, (**d**) PS80, and (**e**) PS100.

XRD Rietveld refinement was conducted using Maud software. The relative quantities of each mineral in the samples and their residues are shown in Figure 2. The contents of Quartz and Hercynite in the biochar samples increased with the amount of SS and RM added. The relative contents of the mineral products determined by Q-XRD analysis are shown in Figure 2. The SIG value is close to 1.5 and Rwp < 10 s the fitting results were significant and reliable [30].

Hercynite is the main iron-containing mineral product in PS80 and PS100, and its maximum content reached 4.31% in PS80. Ferric oxide may be reduced either with carbon or with organics; therefore, PS80 yielded more Fe(II) phase when compared with PS100. Both Magnetite (Fe_3_O_4_) and Hematite (Fe_2_O_3_) phases in the pyrolysis products increased significantly with the increasing SS and RM content, while the zero-valent iron was not identified. According to the Fe content in the raw materials, there is a possibility that amorphous Fe could exist in the pyrolysis products.

(2) $${3\mathrm{Fe}}_{2}O_{3}+C\overset{600–800℃}{\to }{2\mathrm{Fe}}_{3}O_{4}+\mathrm{CO}$$

(3) $${\mathrm{Fe}}_{3}O_{4}{+\mathrm{Al}}_{2}O_{3}\overset{800℃}{\to }{\mathrm{FeAl}}_{2}O_{4}+{\mathrm{Fe}}_{2}O_{3}\;$$

(4) $${\mathrm{FeSiO}}_{3}{+\mathrm{Al}}_{2}O_{3}\overset{800℃}{\to }{\mathrm{FeAl}}_{2}O_{4}+{\mathrm{SiO}}_{2}\;$$

(5) $$\mathrm{FeO}+{\mathrm{Al}}_{2}O_{3}\overset{800℃}{\to }{\mathrm{FeAl}}_{2}O_{4}\;$$

The values of Gibbs free energy changes, ΔG^o^, were negative for reactions (3) and (4), indicating that the reaction of iron-containing minerals with Al_2_O_3_ to form Hercynite could proceed spontaneously.

### 3.3. Tetracycline Removal by Sludge Biochar

#### 3.3.1. Tetracycline Removal with Different Sludge-Waste Content, Biochar Dosage, and pH

**Figure 3:**
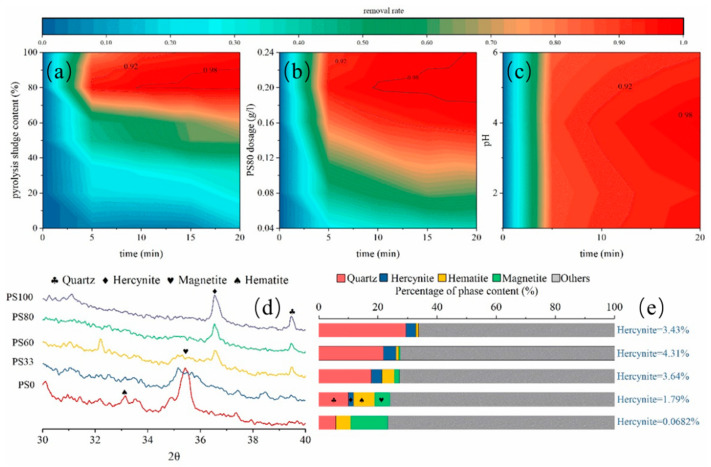
The effect of (**a**) different sludge-derived biochar and (**b**) different dosages of PS80 and (**c**) different initial pH on TC removal; (**d**) XRD patterns and (**e**) mineral constituents of different sludge-derived biochar.

The effect of biochar with different sludge contents on TC degradation is shown in Figure 3a. It can be seen that with the increase in sludge content in biochar, the degradation efficiency presents a rising trend. The degradation efficiency of the PS80 sample reached 95% after 5 min and 99.1% after 20 min. This may be because when the sludge content is low, it is difficult to form the FeAl_2_O_4_ phase due to the insufficient reduction of gas in the pyrolysis process. Compared with the PS80 sample, the degradation efficiency of PS100 for TC decreased, suggesting that the FeAl_2_O_4_ phase in pure sludge pyrolysis products decreased. In other words, steel slag and red mud provide iron oxide and aluminum oxide to form the FeAl_2_O_4_ phase during the co-pyrolysis process with sludge. This also indicates that adding appropriate amounts of steel slag and red mud in the dewatering process can not only help to improve the dewatering performance of sludge but also play an important role in the formation of biochar with catalytic function.

The effect of different PS80 dosages on TC degradation efficiency is shown in Figure 3b. With the increase of the dosage from 0.04 g/L to 0.20 g/L, the degradation efficiency was significantly improved, and the degradation efficiency was nearly 99% at 10 min.

Figure 3c illustrates the effect of the different pH levels of solutions on the TC degradation efficiency. The results indicate that acidic solution has little effect on degradation efficiency. With the decrease in pH levels from 6 to 1, the degradation efficiency increased slowly, which may be due to the acidic environment promoting the dissolution of a small amount of Fe^2+^ from the Fe^0^ and iron–carbon compound (Fe_0.95_C_0.05_), forming a homogeneous reaction [21,28]. Moreover, the negatively charged biochar has an electrostatic repulsion to anionic TC^−^ and TC^2−^ at high pH levels [31], leading to decreasing sorption performance.

#### 3.3.2. Tetracycline Removal Mechanism

To investigate the role of hercynite in biochar, the advanced oxidation effect of Fe-containing phases on the removal efficiency of TC by PS80 and PS0 with H_2_O_2_ or without H_2_O_2_ was detected. A pseudo-first-order model (PFO), pseudo-second-order (PSO) model, and intra-particle diffusion model (IP) were used for the clinical fitting of the data through the linear form using Equations (6)–(8):

(6) $$\ln(Q_{e}-Q_{t})=\ln Q_{e}-k_{1}t$$

(7) $$\frac{t}{Q_{t}}=\frac{1}{k_{2}Q_{e}^{2}}+\frac{t}{Q_{e}}$$

(8) $$Q_{t}=k_{3}t^{1/2}$$

where *Q_e_* (mg g^−1^) and *Q_t_* (mg g^−1^) are the amount of TC on PS80 at any time *t* (min) and equilibrium, respectively, and *k*_1_ (min^−1^), *k*_2_ (g·mg^−1^·min^−1^), and *k*_3_ (mg·g^−1^·min^−1/2^) are the rate constants. Experimental data were fitted and the model parameters were listed in Table 2. It could be observed that, for the PS80-H_2_O_2_ sample, the coefficient of correlation (R^2^) is higher for the PSO (R^2^ = 1.0000) than for the PFO (R^2^ = 0.9963). Conversely, the R^2^ values were higher for the PFO (R^2^ = 0.9932) than for the PSO (R^2^ = 0.9871) for the PS80 sample. It indicates that the PSO was more appropriate for describing the adsorption behavior of TC onto PS80-H_2_O_2_, and the PFO fit better for the adsorption behavior of TC onto PS80. The PFO and PSO models describe the kinetics of the adsorption rate for liquid–solid systems [32], which are based on the adsorption capacity of the solid. Therefore, the uptake of TC onto PS80 is similar to the phenomenon of physisorption, while the uptake of TC onto PS80 combined with H_2_O_2_ is more similar to the chemisorption process.

According to the fitting results, the equilibrium adsorption quantity *Q_e_* for PS80 with H_2_O_2_ (100.13 mg g^−1^) was much higher than for PS80 without H_2_O_2_ (72.63 mg g^−1^), implying a better adsorption performance for PS80-H_2_O_2_. It was demonstrated that the Fe/Al atoms in the [Fe^2+^-Al^3+^] unit on the surface of the FeAl_2_O_4_ phase could react with H_2_O_2_ to produce the ·OH and OH^−^ group. It is noted that the uptake of TC onto PS80-H_2_O_2_ is also a physisorption-controlled reaction, since the diffusion process of the TC reaching the surface pore of the biochar is slower than the homogeneous Fenton reaction between the adsorbed TC and the ·OH generated from the surface of biochar.

**Figure 4:**
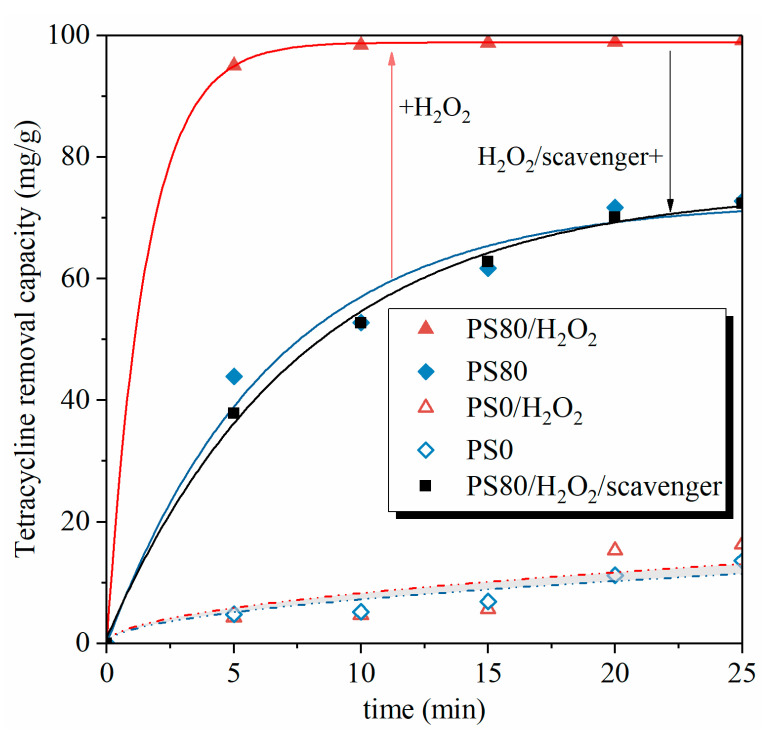
TC removal capacity by PS80 and PS0 with and without H_2_O_2_.

As shown in Figure 4, the removal capacity of TC by PS0 with or without H_2_O_2_ differences in the TC removal efficiency is not significant, mainly due to the main Fe-containing phases Hematite and Magnetite in the PS0 presented in Figure 3d,e. The IP fitted quite well for the TC removal process by PS0 and PS0-H_2_O_2_, indicating that the film diffusion process was the main rate-controlling mechanism for this adsorption system at 0–25 min; it involves the slow movement of the TC from the boundary layer to the RMSS bend’s surface. As can be seen in Table 2, the TC removal rates for PS0 and PS0-H_2_O_2_ were approximately equal. This means that there was no obvious Fenton reaction that occurred in the PS0 groups.

The chemical and mineralogical compositions of varieties of sludge are reported in Table 3. The alumina content was increased with the increase in sludge amounts, and the iron content was decreased at the same time, due to the high Al and the low Fe content in the sludge. The Al_2_O_3_/Fe_2_O_3_ value in PS80 was 2.314, which is quite close to the Al/Fe stoichiometric ratio of FeAl_2_O_4_ and occurs in much higher FeAl_2_O_4_ content levels in the biochar.

**Figure 5:**
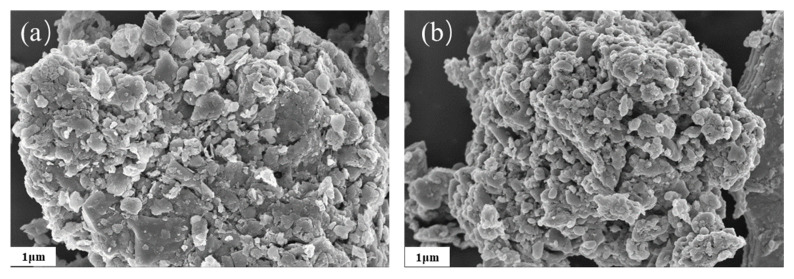
SEM of biochar (**a**) PS0 and (**b**) PS80.

According to the waste materials bloating theory proposed by Riley, the silica and alumina and the fluxing content (i.e., the sum Fe_2_O_3_ + MgO + CaO + Na_2_O + K_2_O) could be used to predict the bloating attitude [33]. The value of Fe_2_O_3_ + MgO + CaO + Na_2_O + K_2_O/(Al_2_O_3_ + SiO_4_) ratio (K) decreased with the increase in the sludge amounts and was inversely proportional with the expansion. SEM observations demonstrated a good pore structure in the PS80 compared with the PS0 (Figure 5). Both samples showed a coacervate that was loose, sponge-like, and permeated with flaky minerals. The coacervate in PS80 was made up of smaller sized particles; most pores had diameters of less than 100 nm. These features could explain the higher removal efficiency of TC by PS80.

PS80 had a better pore structure; biochar could absorb the TC, and then the TC on the biochar surface would be degraded by the FeAl_2_O_4_ through the heterogeneous Fenton reaction. Research shows that heterogeneous Fenton reactions initiated by the bound iron phases (Fe_3_O_4_ and FeAl_2_O_4_) on the surface of the biochar could increase the amount of radical hydroxyl radical (·OH) generation. To confirm the role of the ·OH on the adsorption, 4 mM tertiary butanol (TBA) was added in the PS80-H_2_O_2_ sample as the free radical scavenger. As can been seen in Figure 4, there were no significant differences between the adsorbing capacity of the PS80 and the PS80-H_2_O_2_/scavenger. Furthermore, the PFO fitted the experimental data well when compared with the PSO (Table 2), indicating that the adsorption behavior of TC onto the PS80-H_2_O_2_/scavenger was back to the physisorption when the ·OH was scavenged by TBA. The mutual promotion effect of FeAl_2_O_4_/biochar for TC removal in this research can be concluded to be as the graphical abstract depicted. The adsorption of TC by biochar could reduce the diffusion distance between ·OH and TC, thus enhancing the heterogeneous Fenton reaction rate. On the other hand, the degradation of TC by FeAl_2_O_4_ released the adsorption sites on the biochar surface and was thus beneficial to the heightening of the adsorption capacity of biochar. The Al_2_O_3_/Fe_2_O_3_ value and the micro-nano pore structure play a key role in this process.

**Figure 6:**
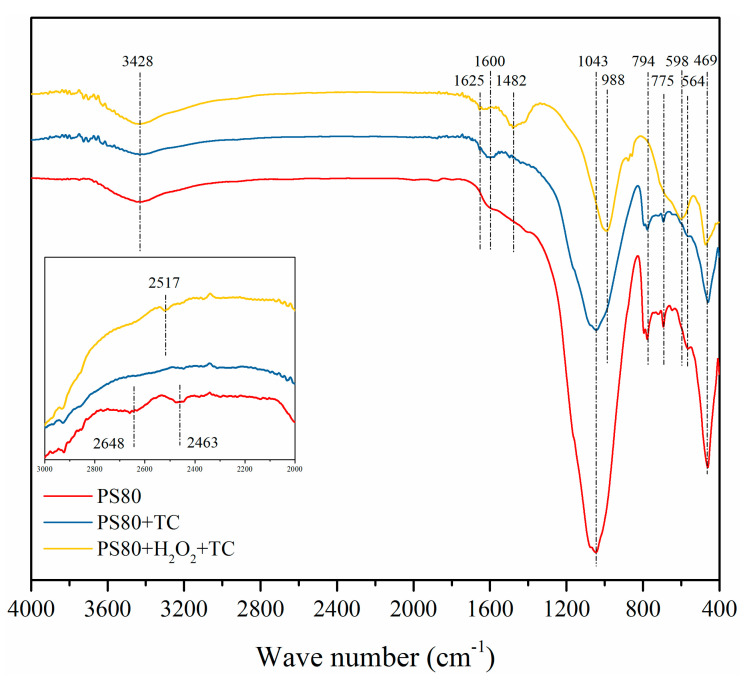
FTIR spectra of the PS80, PS80+TC, and PS80+H_2_O_2_+TC samples.

To reveal the mechanisms involved in the TC removal process, FTIR analyses of the PS80 before and after sorption of TC at 25 min with H_2_O_2_ (PS80-H_2_O_2_-TC) and without H_2_O_2_ (PS80-TC) were performed and are shown in Figure 6. Functional groups of PS80, PS80-TC, and PS80-H_2_O_2_-TC are presented in Table 4. As can be seen in the depiction of the waste and sludge-derived biochar PS80, the strong peak located at 1043 cm^−1^ was assigned to C–O-C stretching vibration and Si-O bonds, which are found in most silicon-rich solid wasted-derived biochar [34]. The bands around 794 cm^−^^1^, 775 cm^−^^1^, and 598 cm^−^^1^ corresponded to the Al-O bending vibration and Fe-O bending vibration, indicating the formation of Fe oxides or Fe-O/Al-O complexes on the surface of the biochar during the pyrolysis process.

For the PS80-TC sample, the bands appearing at 1600 cm^−1^ could be assigned to the C=O stretching at ring A in TC. Compared to PS80, the peaks at 2648 cm^−1^ and 2463 cm^−1^ disappeared in PS80-TC and there were no other peak shifts, indicating that TC was bound to the PS80 surface controlled by physical adsorption. In PS80-H_2_O_2_-TC, the peaks of 1600 cm^−1^, 1043 cm^−1^, and 564 cm^−1^ shifted to 1625 cm^−1^, 988 cm^−1^, and 598 cm^−1^, indicating that C=O containing functional TC and Fe-O groups in PS80 may participate in the adsorption process. The comparison of the removal capacity for TC with other materials shows the obvious advantages of PS80 (Table 5), indicating a good application prospect.

## 4. Conclusions

In this study, a micro-nano pore structure biochar (PS80) derived from the co-pyrolysis of RMSS and sludge was synthesized, and its TC removal mechanism was clarified. Indeed, the sludge dewatering performance was improved after conditioning with RMSS, contributing to the destruction of the flocculent structure of sludge and the provision of a porous skeleton structure and Fe^3+^ by weak acid-modified RMSS. The presence of the FeAl_2_O_4_ phase in the co-pyrolysis biochar was confirmed by QXRD and FTIR. Biochar PS80, produced by a co-pyrolysis comprising 20% RMSS and 80% sludge, has a maximum TC removal efficiency of 99.1% combined with H_2_O_2_ at a TC dosage of 40 mg/L, a 25 min contact time, and a temperature of 25 °C. The removal process fits the pseudo-second-order kinetics model, suggesting that the process was controlled by chemisorption; the movement of the TC from the liquid phase onto the PS80 without H_2_O_2_ was found to be a physisorption process. The relevant adsorption mechanism is as follows: (1) The RMSS changes the Fe/Al and the Fe_2_O_3_ + MgO + CaO + Na_2_O + K_2_O/(Al_2_O_3_ + SiO_4_) ratio of the blend to within a suitable range, thus inducing the formation of FeAl_2_O_4_ in the biochar. (2) Anionic TC^−^ and TC^2−^ enters the biochar pores to deposit on the surface of the adsorbent at a pH level from 2 to6. (3) FeAl_2_O_4_ on the surface of the biochar increases the amount of ·OH generation to degrade the TC. (4) The degradation of TC by FeAl_2_O_4_ releases the adsorption sites on the biochar surface, heightening the adsorption capacity of biochar. We propose that RMSS-sludge-derived biochar can be used as a cost-effective adsorbent to remove TC from wastewater.

## Figures and Tables

**Table 1:** Main inorganic chemical compositions of RS, SS, and RM (wt %).

Constituents	CaO	MgO	SiO_2_	Fe_2_O_3_	Al_2_O_3_	MnO	P_2_O_5_
SS	41.09	19.40	15.68	12.21	6.36	2.36	1.10
RS	2.69	1.48	55.74	5.86	23.62	-	5.13
RM	0.96	0.17	15.33	36.21	25.21	0.03	0.16

**Table 2:** Parameters from the kinetic modeling of Tetracycline on biochar.

Sample	Pseudo-First-Order Model			Pseudo-Second-Order Model			Weber Model	
	R^2^	*K*_1_ (min^−1^)	Q_e_(mg·g^−1^)	R^2^	*K*_2_ (g·mg^−1^·min^−1^)	Q_e_(mg·g^−1^)	R^2^	*K*_3_ (g·mg^−1^·min^−1^)
PS80-H_2_O_2_	0.9963	0.6467	98.87	1.0000	4.4258	100.13	-	-
PS80	0.9932	0.1538	72.63	0.9871	0.1740	91.15	-	-
PS80-H_2_O_2_ /scavenger	0.9995	0.1297	74.80	0.9982	0.0013	95.86	-	-
PS0-H_2_O_2_	-	-	-	-	-	-	0.8985	0.0262
PS0	-	-	-	-	-	-	0.9340	0.0229

**Table 3:** Chemical Composition and Firing Behavior of red mud-steel slag-sludge.

	PS0	PS33	PS60	PS80	PS100
Al_2_O_3_	0.1579	0.1837	0.2049	0.2205	0.2362
Fe_2_O_3_	0.2421	0.1815	0.1320	0.0953	0.0586
K *	0.5052	0.9708	0.5415	0.3265	0.1635
A/F	0.6520	1.0119	1.5520	2.3141	4.0307
yields	0.9876	0.9202	0.8844	0.8215	0.7730
BET (m^2^/g)	4.36	28.66	40.17	46.40	37.52

*: K = Fe_2_O_3_ + MgO + CaO + Na_2_O + K_2_O/(Al_2_O_3_ + SiO_4_).

**Table 4:** Functional groups of biochar before and after TC sorption.

Wave Number (cm^−1^)			Assignment
PS80	PS80-TC	PS80-H_2_O_2_-TC	
3428	3428	3428	-OH stretching
2648			
2463			
		2517	
		1625	stretching vibration of -OH
	1600		C=O bond in the TC [35]
		1428	
1043	1043		C-O-C stretching vibration [31] and Si-O bonds
		988	
794	794		Al-O bending vibration
775	775		Al-O bending vibration
		598	Fe-O bonds
564	564		Fe-O bending vibration [36,37]
469	469	469	Si-O-Si bonds

**Table 5:** Comparison of the removal capacity for TC with other materials reported in the literature.

Materials	C_0_ (mg/L)	Removal Rate (%)	References
PFSC-900/PMS	40	90.10	[38]
MBC	20	98.70	[39]
g-C_3_N_4_/BC/Bi_25_FeO_40_	20	92.20	[40]
MKBC	50	84.15	[41]
MPBC	20	98.77	[42]
PS80	40	99.10	This study

## Data Availability

The data presented in this study are available on request from the corresponding authors.
